# Transendoscopic Ventriculocordectomy Using Monopolar Electrosurgical Instrumentation for Conjunctive Treatment of Laryngeal Hemiplegia in Horses: 24 Cases (2017–2019)

**DOI:** 10.3389/fvets.2021.628410

**Published:** 2021-03-01

**Authors:** McKenna K. Caspers, Chris D. Bell, Dane M. Tatarniuk

**Affiliations:** ^1^Department of Veterinary Clinical Sciences, Iowa State University, Ames, IA, United States; ^2^Elders Equine Veterinary Services, Winnipeg, MB, Canada

**Keywords:** ventriculocordectomy, equine, electrocautery, electrosurgery, recurrent laryngeal

## Abstract

**Objective:** The objective of this study is to evaluate the safety, efficacy, and owner satisfaction following electrosurgical ventriculocordectomy (EVC), in conjunction with prosthetic laryngoplasty, in equine clinical cases affected with left- or right-sided recurrent laryngeal neuropathy.

**Methods:** Retrospective data analysis of clinical signalment, surgery, athletic outcome, intra- and postoperative complications, and postoperative examinations from clinical cases wherein EVC was performed in conjunction with traditional prosthetic laryngoplasty from one practice. Owners were contacted by phone or email for a follow-up questionnaire.

**Results:** Twenty-four horses underwent unilateral EVC, performed transendoscopically under sedated restraint, using monopolar electrosurgical instrumentation successfully. One horse experienced excessive intraoperative hemorrhage. No horses demonstrated postoperative complications. Twenty cases had a history of increased airway noise prior to surgery. In 15 of these cases (15/20, 75%), the airway noise was reported as fully improved post-surgery. Eighteen cases had a history of exercise intolerance prior to surgery. In 15 of these cases (15/18; 83%), the exercise intolerance was reported as resolved.

**Conclusion:** EVC, in conjunction with prosthetic laryngoplasty, can contribute to improvement of RLN symptoms and aid in the effective return to athletic work. Performing transendoscopic ventriculocordectomy with monopolar electrosurgical instrumentation provides comparable clinical outcomes to traditional techniques using a diode laser or direct excision via laryngotomy.

## Introduction

Recurrent laryngeal neuropathy (RLN) is an idiopathic upper airway disorder commonly seen in horses. Previous prevalence has been reported at 2.8–8% in Thoroughbreds and upwards of 35% in draft breeds (1–3). Paralysis of the associated arytenoid, secondary to RLN, results in a decreasing size of the rima glottidis. As inspiratory negative pressure increases during work, exercise intolerance and airway noise can develop secondary to the obstructing arytenoid and impede athletic function (4).

Ventriculocordectomy (VC) is a surgical procedure that increases the ventral diameter of the rima glottidis, thus decreasing both impedance and airway turbulence responsible for noise production and occlusion of the airway (5, 6). Prosthetic laryngoplasty, in combination with a VC, is currently considered to be the surgical treatment of choice for horses affected with RLN (4).

VC can be performed as a standing procedure under sedation or under general anesthesia via a laryngotomy approach (5, 7, 8). The procedure can also be performed in the standing, sedated horse using transnasal endoscopic guidance with excision performed via use of a diode laser or a Nd:YAG laser passed through the biopsy portal of the endoscope (9, 10). Recently, the use of a monopolar electrosurgical triangle-tip knife has been validated as a safe and effective instrument for performing endoscopic VC in healthy horses (11). A monopolar electrosurgical triangle-tip knife has the clinical advantages of using equipment often more readily available in hospitals and is more cost-effective compared to diode laser equipment (11). Diode laser units can be anywhere from 5 to 15 times the cost of the cautery unit, and diode laser fibers are two to three times the cost of the triangle-tip knife (11). Additionally, the triangle-tip knife can be used up to at least nine consecutive VC surgeries (11).

The objective of this study is to evaluate the clinical use, patient outcome/complications and owner satisfaction following electrosurgical ventriculocordectomy (EVC) when performed in conjunction with prosthetic laryngoplasty, in clinical cases affected with left- or right-sided RLN. We hypothesize that VC performed using the monopolar electrosurgical triangle-tip knife, when performed concurrent with prosthetic laryngoplasty, will contribute to clinical improvement of RLN symptoms and an effective return to athletic work similar to outcome following established VC surgical techniques combined with prosthetic laryngoplasty.

## Materials and Methods

Medical records were searched to identify horses that presented with unilateral laryngeal hemiplegia and that were treated with prosthetic laryngoplasty and conjunctive EVC at one hospital. Preoperative static video endoscopy was performed to confirm the diagnosis of RLN for all cases. The medical record was evaluated for data regarding patient signalment, presenting complaint by owner, discipline/intended use at time of surgery, concurrent airway abnormalities (in addition to RLN), Havemeyer grade of RLN (12), results of other imaging modalities, pre- and post-surgical management of the patient, and any intra- or postoperative complications.

All procedures were performed by one board-certified large-animal surgeon. All cases of EVC were performed as a unilateral procedure on the affected side. No preoperative medications were administered. Standing sedation was achieved with an intravenous injection of 5 mg of detomidine (Dormosedan; Zoetis, Parsippany, NJ) followed by a continuous rate infusion of 20 mg of detomidine in a 500-mL saline bag, titrated to effect (approximately 0.005 to 0.01 mg/kg/h). Lidocaine (Lidocaine 2%; VetOne, Boise, IA) (~20–40 mL) was administered topically on the nasal passageway, arytenoids, and vocal cords through the endoscopic biopsy channel, using endoscopic visualization, ~5 min prior to surgery.

**Figure 1:**
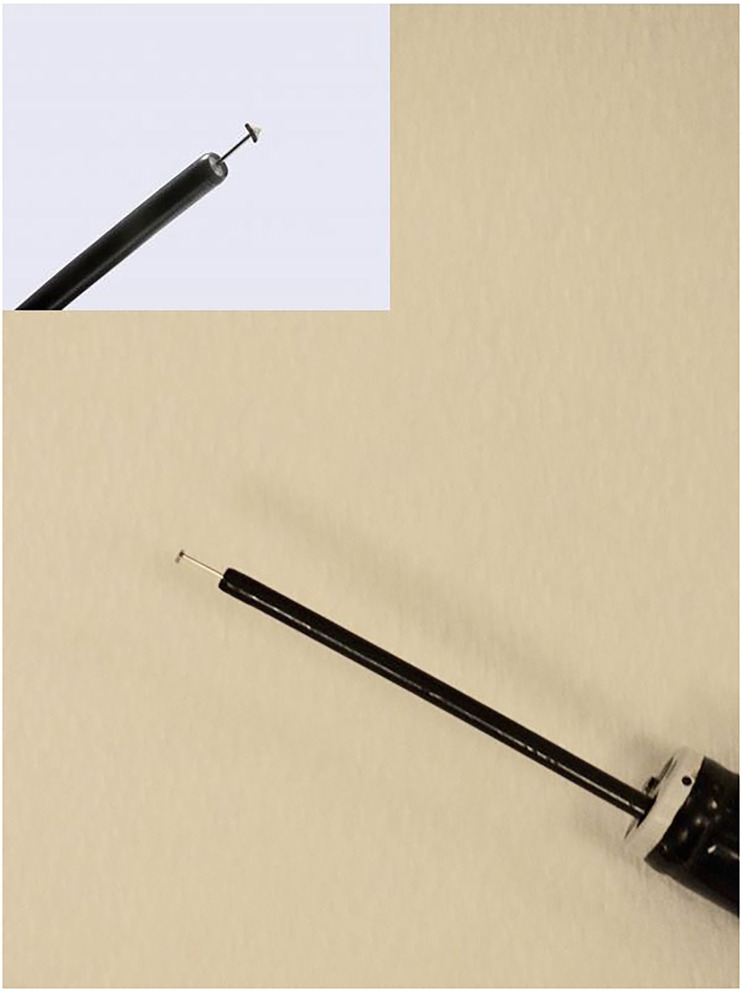
Image of the monopolar triangle-tip electrosurgical knife passed through a biopsy portal of the endoscope. The triangle-tip knife has three sides to aid in creating incision and dissection of tissue without the need to rotate the knife within the endoscope channel.

**Figure 2:**
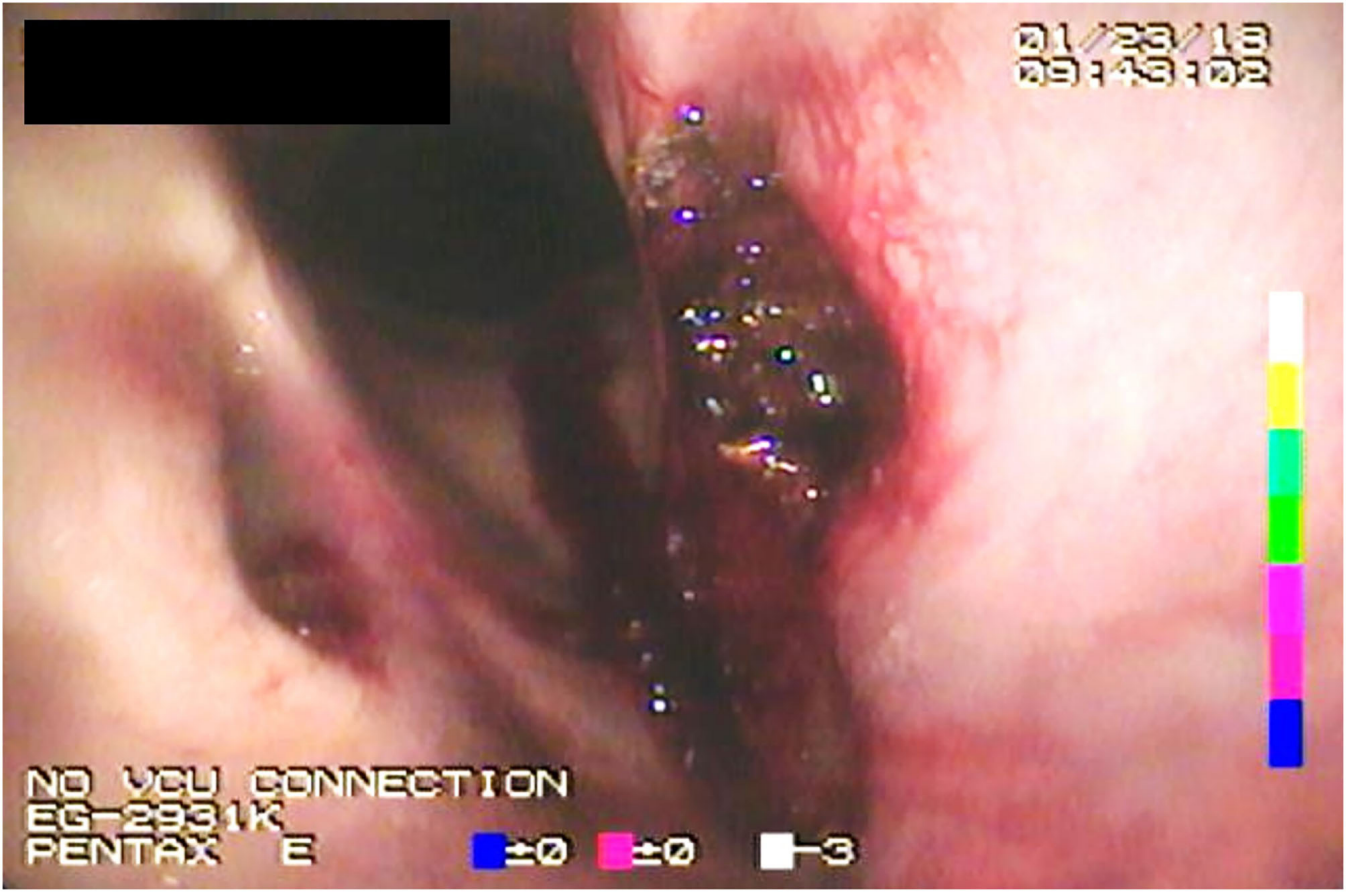
Endoscopic image acquired immediately postoperatively following electrosurgical ventriculocordectomy.

Monopolar conduction was achieved by the use of an adhesive hydrogel contact pad applied to the neck, lip, or tongue of the horse. Occasionally, hair was clipped to facilitate contact when necessary. Grasping endonasal tracheal (ENT) forceps (Optomed, Bâtiment, France) were passed through the contralateral nasopharynx, which were used to grasp and evert the saccule mucosa within the left laryngeal ventricle. The ENT forceps were 65 cm in length, 3.5 mm in diameter, and semi-rigid, which allowed for bending of the forceps to the appropriate curvature (~30°) of the upper airway. The ENT forceps tips have horizontal alligator jaws, and the handle has a ratchet that allows locking of the jaws. A monopolar electrosurgical triangle-tip knife (Olympus America, Center Valley, PA) attached to a cautery unit (SurgiStat SURGII-20; Coviden-Medtronic, Minneapolis, MN) was passed through the biopsy channel of a 1-m videoendoscope (Evis Exera III; Olympus America, Center Valley, PA) and applied in contact fashion at a setting of 25 W for both coagulation and cut modes of use. The triangle-tip knife has a working length of 165 cm and a cutting knife length of 4.5 mm and uses a minimum channel of 2.8 mm (Figure 1). The videoendoscope has a diameter of 8 mm and a biopsy channel diameter of 3 mm. A combination of coagulation, coagulation and cut, or a blended cut setting is possible to be used to perform the procedure. Surgically, the laryngeal saccule was excised in a dorsal-to-ventral direction with removal of ~70% of the saccule based on surgeon discretion. After the saccules were excised, the vocal cord was resected in a dorsal-to-ventral direction in a half-moon, crescent pattern (Figure 2). Excision in a dorsal-to-ventral direction assisted in avoiding hemorrhage, originating from the vascular region of the ventral commissure, which can obscure endoscopic visualization.

A standing left laryngoplasty via placement of two individual sutures using five braided polyester (Ethibond Excel®, 7.0 metric; V-37, Ethicon Inc., Cincinnati, OH) with no additional implants was subsequently performed following EVC. Postoperative medications included flunixin meglumine (IV, 1.1 mg/kg SID for 4 days), gentamicin (IV, 6 mg/kg SID for 3 days), and procaine penicillin (IM, 22,000 IU/kg SID for 3 days). Hospitalization time postoperatively was 4 days for all cases. Postoperative recommendations included confinement to stall rest for 4 weeks, with hand-walking only. After 4 weeks, horses could be turned out into a small paddock for another 4 weeks. No forced work or exercise was recommended for 8 weeks post-surgery. In addition, it was recommended to dampen hay and feed off the ground.

Owners were contacted by phone or email for a follow-up questionnaire. Owners were asked if the horse had any persisting or recurring exercise intolerance, if it had respiratory noise during exercise, if there were any limitations returning to the horse's intended use (chosen discipline at the time of surgery), if there were any long-term complications following the surgery, if the owner was satisfied with the surgery (yes/no), if the horse was still alive, and if the horse was available for follow-up upper airway endoscopy. In select cases, if the horse was available for follow-up, repeat upper airway endoscopy was performed and findings were documented.

## Results

Electrosurgical VC was successfully performed in 24 horses. This included 14 Percherons, four Clydesdales, three Thoroughbreds, two Quarter Horses, and one Warmblood. There were five mares and 19 geldings. Age ranged from 3 to 13 years, with a mean age of 5.3 years (standard deviation, 2.5 years). Grade of laryngeal hemiplegia ranged from 1 to 4. Grouped, there was one horse as grade 1, eight horses as grade 3, and 15 horses as grade 4. Eighteen of the horses were used for driving/show hitch, two for barrel racing, two for show jumping, and two for thoroughbred racing. Fourteen horses had both exercise intolerance and increased respiratory noise noted as a presenting complaint by the owners, while six had only exercise noise, and the remaining four cases exhibited only exercise intolerance.

Intra-operatively, one horse (1/24, 5%) experienced hemorrhage from the VC site (~300 mL), partially obscuring and prolonging the endoscopic procedure. No complications were noted in any of the cases during the immediate postoperative hospitalization, and no horses underwent a second procedure or revision surgery during the immediate postoperative period for removal of further tissue. Follow-up with the owner was available in all 24 cases.

Prior to surgery, exercise intolerance was noted in 18 of the 24 cases. Of the 18 cases with the original presenting complaint of exercise intolerance, exercise intolerance was resolved in 15 cases (15/18; 83%), failed to improve in two cases (2/18; 11%), and was unknown at the time of follow-up in one case. Twenty cases had a history of increased airway noise prior to surgery. In 15 of these cases (15/20, 75%), the airway noise was reported as fully improved, and in one horse (1/20, 5%), airway noise was described as improved but not fully resolved. There were four cases (4/20, 20%) where airway noise improved following surgery, but then recurred. In cases of airway noise recurrence, duration to recurrence (as estimated by owner) ranged from 2 months to 1 year, with a mean time to recurrence of 7 months (standard deviation, 1.5 months).

Only three of the 24 cases had a follow-up endoscopy available for veterinary evaluation. This included two Percheron geldings used for show hitch competition and one Quarter Horse used for barrel racing. In one Percheron, right laryngeal hemiplegia was noted. This had been previously documented prior to surgery and the right laryngeal hemiplegia had progressed in causing partial obstruction of the rima glottidis. In the second Percheron, a partial arytenoidectomy was subsequently performed due to a failed left prosthetic laryngoplasty. Although loss of arytenoid abduction was noted, the region of the vocal cords and ventricle had a normal post-excision appearance. In the Quarter Horse, dynamic pharyngeal collapse was appreciated on follow-up endoscopy. All three cases had relapse of airway noise.

Long-term upper airway abnormalities or complications following prosthetic laryngoplasty and electrosurgical VC were noted by owners during follow-up conversation in six of 24 cases. This included the cases of right laryngeal hemiplegia, dynamic pharyngeal collapse, and failed tie-back requiring partial arytenoidectomy previously mentioned. Additionally, there was one case of postoperative infection of the prosthetic laryngoplasty site. The remaining two horses were reported to have had exercise intolerance (1) and persisting airway noise (1); however, the reason for lack of improvement in clinical symptoms was unknown. Excluding the case of postoperative infection, the remaining five cases with long-term upper airway abnormalities did not return to their intended athletic use. Development of choke, pneumonia, cough, or dysphagia was not noted for any cases in which follow-up was obtained. At the time of follow-up, 20 cases (20/24; 83%) were alive, and in four cases (4/24, 17%), the horse had transferred ownership by the time of follow-up and therefore the current state of the horse was unknown. All owners expressed satisfaction with the decision to pursue surgical treatment that utilized conjunctive EVC.

## Discussion

This study provides evidence that the electrosurgical triangle-tip knife can be successfully implemented to perform VC, in conjunction with prosthetic laryngoplasty, to contribute to an improvement in exercise tolerance and abnormal noise production, similar to outcome following VC performed via open laryngotomy or via diode laser. Additionally, the procedure is associated with low intra- and postoperative complications, consistent with results identified in pilot research (11).

Previous outcomes reported for airway noise resolution ranged from 73 to 75% following ventriculectomy or VC performed via laryngotomy, in conjunction with laryngoplasty (13, 14). Results from this study are consistent, in that 15/20 (75%) of horses improved in airway noise. Previous positive outcomes reported for exercise intolerance ranged from 69 to 71% (13, 14). Results from this study are also similar, with 15/18 (83%) improving in exercise tolerance. Several factors may affect comparison of our study to previous outcomes, such as a lower overall sample size. Furthermore, both studies were primarily light breed horses (predominately thoroughbred horses (14) or a mixed breed population (13)), which contrasts to this study that was mostly draft horses.

Prior research investigating return to intended use following VC when using either a Nd:YAG or diode laser, in conjunction with a prosthetic laryngoplasty, ranged from 66 to 90% (7, 9). These studies obtained follow-up data through questionnaires posed to the owners, similar to our study methodology. In an investigation focused solely on draft horse breeds, Nd:YAG laser or diode laser VC performed with prosthetic laryngoplasty resolved airway noise and exercise intolerance in 45/68 cases (66%). Additionally, all cases returned to their intended use (9). Results using EVC with prosthetic laryngoplasty are similar, with 15/24 (63%) of horses resolving both airway noise and exercise intolerance and being able to return to intended use.

Specific to draft horses and the effect of traditional VC alone on airway noise, Cramp et al. identified 15/18 (83%) horses improved airway noise sufficiently to resume competition (15). In another study representing a mixture of disciplines (show jumping, fox hunting, flat racing, steeplechase racing, driving, dressage, and pleasure riding) undergoing diode laser VC alone, 20/22 (90%) of horses returned to their intended use, 8/10 (80%) improved in exercise intolerance, and 18/22 (82%) had abnormal airway noise eliminated (7). In comparison, 15/20 (75%) of horses had full resolution of airway noise in our study. It is important to consider that there may be an influence on postoperative airway noise by also performing prosthetic laryngoplasty in conjunction with VC, which is specific to our study.

In this study, EVC was associated with a low intraoperative complication rate (one case, excessive hemorrhage). Postoperative revisions included one case that had right-sided RLN present prior to surgery that ultimately required intervention following initial surgery on the left side, as well as another case that had a failed prosthetic laryngoplasty. In both instances, it is unlikely that the use of the electrosurgical instrument contributed to, or worsened, abnormalities that were present. In comparison, reported complications following diode laser VC used alone included 3/22 developing laryngeal damage, subdivided as one case of postoperative swelling causing respiratory distress, one case developing a granuloma noted at 4 weeks postoperatively, and one case developing mild right arytenoid chondritis that responded to medical management (7). While the sample size of our study is low, and some postoperative complications that may not create clinical signs (such as granuloma formation or asymptomatic arytenoid chondritis) could have been missed due to infrequency of follow-up endoscopy or shorter durations of postoperative hospitalization, the safety profile of EVC with prosthetic laryngoplasty is encouraging. It is also important to note that complication rates following transendoscopic surgery are often related to iatrogenic and inadvertent thermal injury, which may be highly influenced by surgeon experience.

Only three horses had follow-up endoscopy performed. The lack of follow-up endoscopy in this study is likely influenced by the positive outcome in many cases with improvement in clinical symptoms and athletic performance, thereby not requiring follow-up examinations. To this point, there were only four out of 24 cases in which airway noise improved and then relapsed. One Percheron re-presented 12 months following initial treatment for recurrence in airway noise. Upon endoscopic examination, the horse had progressed in previously noted right laryngeal hemiplegia. Due to previous left prosthetic laryngoplasty and electrosurgical VC performed, the right laryngeal hemiplegia was subsequently treated with EVC alone to avoid subsequent risks of aspiration following a bilateral prosthetic laryngoplasty procedure. Etiology of right RLN in this case is unknown, but it can be speculated that left-sided prosthetic laryngoplasty and EVC improved air flow initially, and as the paralysis on the right side progressed, allowing airway noise to relapse. In the second Percheron with follow-up endoscopy, a failed left prosthetic laryngoplasty was noted and subsequently treated with partial arytenoidectomy. The failed prosthetic laryngoplasty is likely unrelated to the instrumentation used for VC, as this is a well-recognized potential complication of prosthetic laryngoplasty. Following partial arytenoidectomy, this horse returned to intended use. The third case (Quarter Horse used for barrel racing) developed recurrence of airway noise 3 months following surgery. Following dynamic endoscopy, dynamic pharyngeal collapse was diagnosed. It is unknown whether EVC could have influenced development of dynamic pharyngeal collapse in this case. The etiology of the fourth case of relapsed airway noise is unknown, as follow-up endoscopy was never pursued.

There are several important limitations to consider when interpreting results of this study. EVC was performed in conjunction with prosthetic laryngoplasty, which makes the intervention difficult to assess in relation to how much improvement this specific instrumentation provides when evaluating exercise tolerance. It has been previously established that VC, in general, contributes to improvement in airway noise more substantially than prosthetic laryngoplasty (5). The comparable results noted in this study compared to prior literature is plausible to support. The electrosurgical triangle-tip knife is safe for performing VC based on outcomes described in a pilot study (11) and the technique itself is performed in the same fashion as transendoscopic diode VC. Other limitations include the fact that our population was heavily skewed toward draft horses that were used for pulling or show hitch competition, which may positively affect the return to intended use. It is reasonable to expect that racing disciplines provide a greater challenge to the success of an upper airway surgery. Although limited in sample size, there were two horses engaged in Thoroughbred racing and one horse engaged in barrel racing that had a positive outcome following prosthetic laryngoplasty and EVC. Recall bias is another important limitation of retrospective studies using client follow-up for data collection. Relying on the owner's assessment of long-term outcomes postoperatively is not as accurate or as consistent compared to follow-up veterinarian assessment. Due to the clinical nature of this retrospective study, there were no objective measures of outcome such as measurement of rima glottidis diameter, measurement of airway noise or turbulence, or objective measures of performance success (placements, race times, or monetary earnings).

In conclusion, VC performed using the monopolar electrosurgical triangle-tip knife, in conjunction with prosthetic laryngoplasty, can contribute to improvement of RLN symptoms in clinical cases. Results from this study are in close association with prior studies evaluating clinical outcome following prosthetic laryngoplasty and diode laser VC. All owners were satisfied with the decision to pursue surgical treatment, even though not all cases were deemed successful in resolving clinical abnormalities. Further prospective investigations evaluating a larger cohort of cases or other breeds/disciplines may be beneficial to further elucidate how EVC compares to traditional methods of surgery.

## Data Availability Statement

The raw data supporting the conclusions of this article will be made available by the authors, without undue reservation.

## Ethics Statement

Ethical review and approval was not required for the animal study because it is a clinical case series on veterinary patients with owner consent.Written informed consent was obtained from the owners for the participation of their animals in this study.

## Author Contributions

MC contributed to acquirement of data, data analysis, and final manuscript review. CB contributed to study conception, performance of surgery for clinical cases, acquirement of data, data analysis, and final manuscript review. DT contributed to study conception, data analysis, manuscript preparation, and final manuscript review. All authors contributed to the article and approved the submitted version.

## Conflict of Interest

The authors declare that the research was conducted in the absence of any commercial or financial relationships that could be construed as a potential conflict of interest.
